# Associations between age at first calving and subsequent lactation performance in UK Holstein and Holstein-Friesian dairy cows

**DOI:** 10.1371/journal.pone.0197764

**Published:** 2018-06-13

**Authors:** Neil T. Eastham, Amy Coates, Peter Cripps, Henry Richardson, Robert Smith, Georgios Oikonomou

**Affiliations:** 1 Bishopton Veterinary Group, Mill Farm, Ripon, North Yorkshire, United Kingdom; 2 Department of Livestock Health and Welfare, Institute of Veterinary Science, University of Liverpool, Leahurst Campus, Neston, Wirral, United Kingdom; 3 National Bovine Data Centre, Speir House, Stafford Park 1, Telford, Shropshire, United Kingdom; 4 Department of Epidemiology and Population Health, Institute of Infection and Global Health, University Liverpool, Leahurst Campus, Neston, Wirral, United Kingdom; Gaziosmanpasa University, TURKEY

## Abstract

Lactation records from 396,534 pedigree Holstein and Holstein-Friesian primiparous cows from 6,985 UK milk recorded herds, calving for the first time during the period between the 1^st^ of January 2006 and the 31^st^ of December 2008, were examined in order to determine the associations between age at first calving (**AFC**) and subsequent production, udder health, fertility and survivability parameters. Heifers were grouped by AFC into single month classes ranging from 21 to 42 months. Mixed effects multivariable regression modelling was used for data analysis. Mean and median AFC were 29.1 and 28 months respectively. Within the study, only 48,567 heifers (12.3% of the studied population) calved for the first time at 24 months of age or younger. 162,157 heifers (40.9%) were 30 months or older at their first calving. An increased AFC was associated with increased first lactation milk, fat and protein yields. The lowest predicted mean 305-day yield (6,617kgs; 95% confidence interval (CI): 6,546–6,687 kgs) was recorded for the 21 month AFC class, significantly lower than any other class. The 36 month AFC class had the highest predicted mean (7,774 kgs; 95% CI: 7,737–7,811 kgs). However, an increased AFC was also associated with increased calving interval and increased first lactation somatic cell count (SCC). Animals calving at 21 months had a predicted mean lactation SCC of 72,765 (95% CI: 68427–77378). Animals calving at 36 months of age had a predicted mean lactation SCC of 86,648 (95% CI: 84,499–88,853). Importantly, an increased AFC was also associated with decreased lifetime daily milk yield and decreased likelihood of calving for a second successive time. Animals calving at 22 months of age had a predicted mean daily lifetime milk yield of 15.24 kgs (95% CI: 15.06–15.35); animals calving at 36 months of age had a predicted mean daily lifetime milk yield of 12.83 kgs (95% CI: 12.76–12.91). Our results highlight the importance of achieving a lower age at first calving which was here associated with improved udder health, increased lifetime daily milk yield, improved reproductive performance and increased likelihood of calving for a second time.

## Introduction

Rearing heifer replacements represents a large investment for dairy producers. These animals are key to the future productivity and profitability of the milking herd. A successful transition into the milking herd is vital if producers are to see a return on the investment made during the rearing period. After feed, heifer rearing costs represent the second largest expense on a dairy farm, accounting for approximately 15–20% of the total costs [1]. Poor growth rates, poor health and inappropriate nutrition during the rearing period often lead to increased AFC, not least due to their negative effect on heifer fertility [2].

Conversely, calving too early may be associated with an increased risk of dystocia [3] as well as a reduction in milk yield, milk components and reproductive performance [4]. Alongside an effect on yield and reproductive performance, a number of studies have explored the associations between AFC and other measurable production parameters. Higher AFC has been associated with a higher milk fat percentage [5–7] and lower milk protein percentage [5].

Age at first calving has also been associated with longevity and as a result has a direct effect on productivity and economic return. An economic balance exists between AFC, rearing and associated costs, and subsequent first lactation performance. A reduction of AFC from 26 months to 24 and 22 months increased the difference between milk yield return and rearing costs by $41.5 (£32) and $24 (£19) per heifer respectively [5]. An increase in AFC above 26 months resulted in a reduction in the difference between milk yield return and rearing costs due to an increase in rearing costs against potential milk sales [5].

Within the UK, the industry recognised target for AFC is 24 months, falling in line with published recommendations [8]. This is considered to represent the optimum AFC with regard to maximising productivity whilst avoiding shortfalls in resulting profitability [9]. In reality, this target is rarely achieved in any country and mean AFC ranges from 24.5 to 31 months [10–12]. A recently published UK study showed that the mean within-herd AFC was 29.6 months and a negative association between survival rate of first lactation heifers and increasing AFC was described [13].

Here, using a large dataset, we describe the AFC of UK milk recorded herds and its relationships with subsequent lactation performance (focusing mainly on production, calving interval, and somatic cell counts).

## Materials and methods

Data were provided by the National Bovine Data Centre (NBDC), formerly the Centre for Dairy Information and derived from lactation records compiled by the following major UK milk recording organisations; National Milk Records (NMR), Cattle Information Service (CIS) and United Dairy Farmers (UDF). An initial database (Microsoft Access 2010®) comprised first lactation and following lactations production records for 446,523 pedigree Holstein/ Holstein-Friesian heifers calving in milk recorded herds in the UK during the period between January 1^st^ 2006 and 31^st^ December 2008.

Information relating to identification (animal ID, name), origin (owner ID number, eartag and prefix), and ancestry (herd book number (HBN), breed, sire ID, sire breed, sire name and sire HBN) were available for each heifer alongside lactation number, age at calving (months) and date of calving. The number of days dry and calving interval were listed for those animals having calved for a second or successive time. All animals included in the study were milk recorded in conformance with International Committee for Animal Recording (ICAR) guidelines on recording practice (ICAR, 2012) [14]. All lactation totals for milk yield, milk components and output of somatic cells were calculated by the named recording organisations using the Test Interval Method (TIM) described in section 2.1.4 of the ICAR Guidelines.

Available production data included total milk yield (kg), total fat and protein (kg), days in milk (DIM) and end dates for both 305-day (up to and including 305 days from calving) and complete lactation (up to the cessation of lactation) periods respectively. A mean 305-day SCC was included for each complete lactation.

### Statistical analysis

Sorting, filtering and deletions from the original dataset were made in Microsoft Access 2010® and Microsoft Excel 2010®. Primiparous cows were grouped according to age (months) at the date of first calving. These were divided into 22 monthly classes from 21 to 42 months of age. Animals calving outside this range were excluded from the study as an AFC below 21 months was unlikely to be planned and may have been the result of an abortion or misalliance mating. Primiparous cows calving for the first time over 42 months of age were excluded as they may have previously completed an unrecorded lactation. To explore the associations between AFC and performance in successive lactations, lactations terminated up until the 31^st^ of December 2012 where included in the dataset. Animals for which successive lactation records were not consecutive by lactation number as recorded in the database were removed.

Anomalies in DIM between 305-day and complete lactation records in any one or more lactation resulted in the removal of a number of animals. Exclusion occurred when DIM for 305-day and complete lactation records were not identical for lactations of 305 days or less duration, when DIM for a complete lactation was listed as 305 days or more and 305-day lactation DIM was listed as less than 305 days and lastly when 305-day lactation DIM was listed as greater than 305 days.

When considering first lactation production performance those individuals who failed to complete a first lactation of over 199 days in milk (lactation period) were removed. This is the criterion used by UK milk recording organisations when calculating official herd averages and in doing so removed a significant number of individuals who may have been sold early in the first lactation for continued milk production in non-milk recorded herds. Furthermore, any primiparous cow completing more than 800 days in milk was removed, in order to exclude those animals where the farmer failed to register a successive calving date. The term “qualifying” is used within this study to describe a lactation which meets these criteria.

The relationship between AFC and fertility was examined through the analysis of calving interval (days between first and second calving). Of those animals completing a qualifying first lactation, animals with a calving interval listed as greater than 800 days were removed to exclude those suspected to have completed an unrecorded lactation. Animals where a calving interval was listed as less than 300 days were also removed.

Recorded lifetime daily yield (**RLDY**) was calculated from completed lactation data. The last possible first calving date for any animal recruited to the study was 31 st December 2008. A four-year window up to the end of the study allowed all animals enough time to possibly complete three lactations. An assessment of RLDY against AFC was made on a dataset that was made up of cows which completed three qualifying lactations. Recorded lifetime production (sum of first three lactation yields) was divided by lifetime days to determine the RLDY of each individual.

Mixed effects regression modelling was performed using STATA 13 (®StataCorp LP) in order to explore the associations between AFC class and various different outcome variables. More specifically the following six models were built:

Model 1: First lactation 305-day milk yield was the outcome variable; AFC was fitted as a fixed effect; sire id nested within season, year and farm were fitted as a random effect. Data from 362,923 animals were available for this analysis. The Akaike’s Information Criterion was used in order to evaluate the model’s goodness of fit.
Model 2: First lactation 305-day fat yield was the outcome variable; AFC was fitted as a fixed effect; sire id nested within season, year and farm were fitted as a random effect. Data from 362,923 animals were available for this analysis. The Akaike’s Information Criterion was used in order to evaluate the model’s goodness of fit.
Model 3: first lactation 305-day protein yield was the outcome variable; AFC was fitted as a fixed effect; sire id nested within season, year and farm were fitted as a random effect. Data from 362,923 animals were available for this analysis. Data from 362,923 animals were available for this analysis. The Akaike’s Information Criterion was used in order to evaluate the model’s goodness of fit.
Model 4: Calving Interval was found to be non-normally distributed and was therefore transformed. The following transformation was used: Transformed calving interval = 1/calvinginterval^2^. Transformed calving interval was the outcome variable; AFC was fitted as a fixed effect; sire id nested within season, year and farm were fitted as a random effect. Data from 303,364 animals were available for this analysis. The Akaike’s Information Criterion was used in order to evaluate the model’s goodness of fit.
Model 5: Log transformed first lactation average SCC was the outcome variable; AFC was fitted as a fixed effect; season nested within year and farm were fitted as a random effect. In the case of logSCC when sire id was included in the analysis the model would not converge. Sire id was for this reason removed from this analysis and the random effect of season nested within year and farm was fitted instead. Data from 210,699 animals were available for this analysis. The Akaike’s Information Criterion was used in order to evaluate the model’s goodness of fit.
Model 6: RLDY was the outcome variable; AFC was fitted as a fixed effect; sire id nested within season, year and farm were fitted as a random effect. Data from 216,896 animals were available for this analysis. The Akaike’s Information Criterion was used in order to evaluate the model’s goodness of fit.

A mixed effects logistic regression model was used to calculate the predicted probability of a primiparous cow surviving to the second lactation. The probability of a first lactation animal surviving from lactation one to two (binary variable: 0 = animal did not survive to the second lactation; 1 = animal did survive to the second lactation) was the outcome variable with AFC being fitted as a fixed effect; season nested within year and farm were fitted as a random effect. Data from 396,441 animals were available for this analysis.

## Results

**Fig 1:**
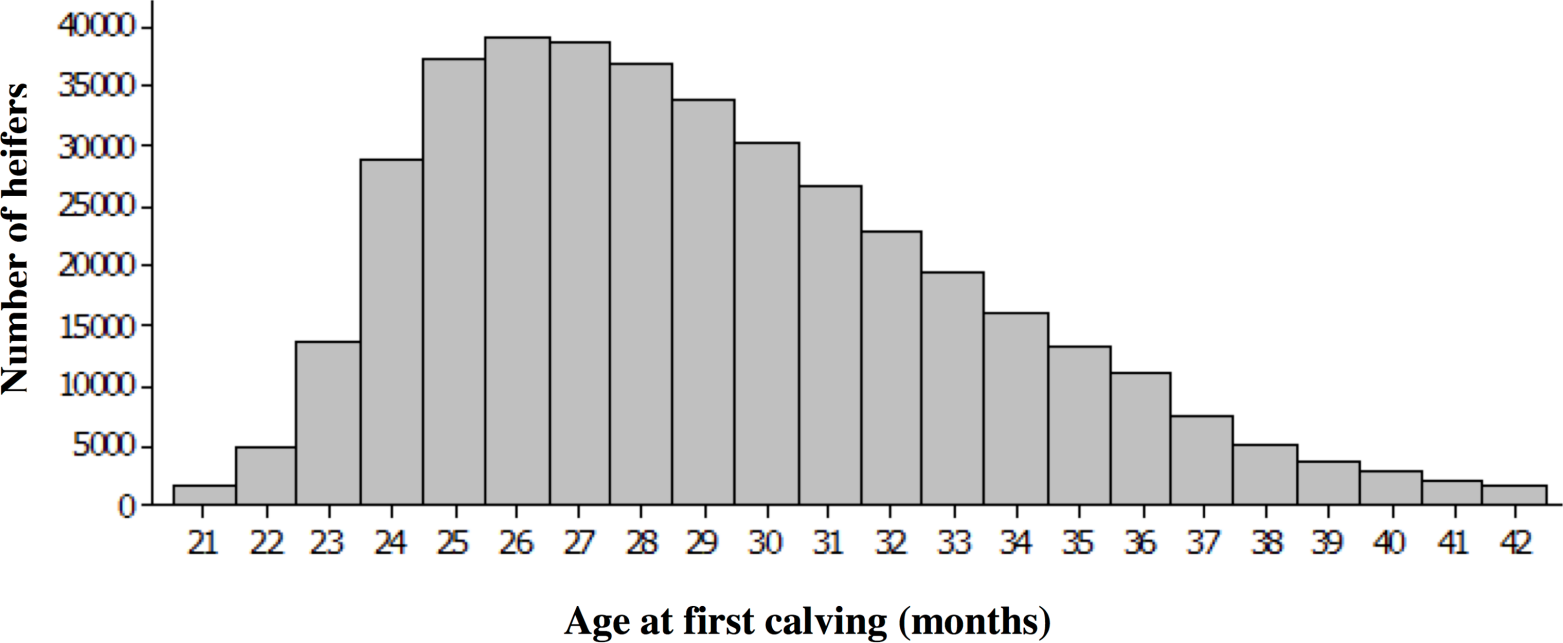
Age at first calving of UK Holstein and Holstein-Friesian heifers. Distribution of age at first calving (AFC) for pedigree Holstein and Holstein-Friesian milk recorded heifers calving in the UK between 01/01/06 and 31/12/08.

Age at first calving distribution is shown in Fig 1. The mean and median AFC across all heifers was 29.1 and 28 months respectively. Of the 396,534 heifers initially included in the study, 48,567 heifers (12.3%) calved for the first time at 24 months of age or younger. 162,157 heifers (40.9%) were 30 months or older when calving for the first time. Of the 6,985 farms in the dataset 849 contributed one heifer, 1,633 contributed 2 to 10 heifers and 4,503 contributed with more than 10 heifers. The average number of heifers per farm was 57 head.

**Fig 2:**
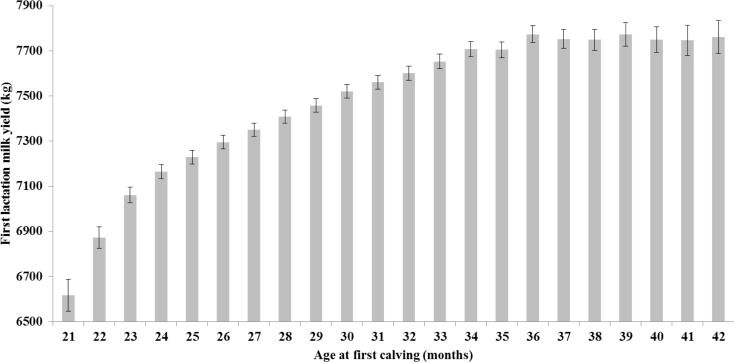
Age at first calving and first lactation milk yield. Predicted means (± 95% confidence intervals) obtained from mixed effects multivariable regression modelling for first lactation (305-day) milk yield (kgs) by different age at first calving (AFC) class.

Fig 2 outlines predicted means (least squares means) (with 95% confidence intervals (**CI**)) of first lactation 305-day milk yield (kgs) obtained from mixed effects regression modelling. The lowest predicted mean 305-day yield (6,617kgs; 95% CI: 6,546–6,687 kgs) is recorded for the 21 month AFC class, significantly lower than any other class. The 36 month AFC class has the highest predicted mean (7,774 kgs; 95% CI: 7,737–7,811 kgs) however this is not significantly higher than the AFC classes 34 to 42 months, inclusive.

**Fig 3:**
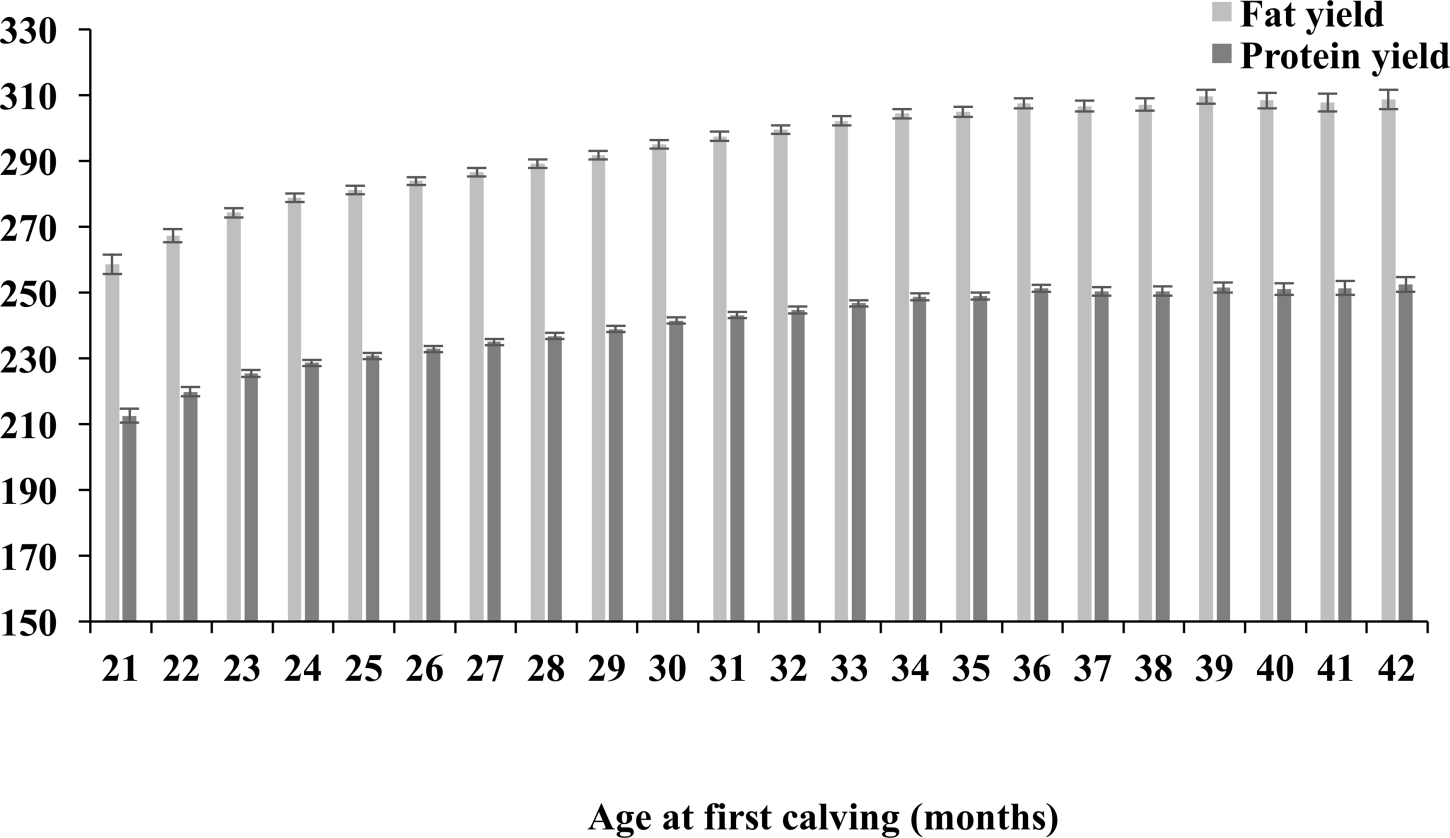
Age at first calving and first lactation fat and protein yield. Predicted means (± 95% confidence intervals) obtained from mixed effects multivariable regression modelling for first lactation (305-day) fat and protein yield (kgs) by different age at first calving (AFC) class.

Trends for 305-day fat and protein yield (kgs) are similar to those predicted for lactation milk yield. Primiparous cows (AFC classes 29 and below) are predicted to produce less fat and protein kgs over a 305-day lactation when compared to those primiparous cows with an AFC of 30 months and greater (Fig 3). Animals calving at 21 months of age had the lowest predicted mean 305-day fat yield (258.49; 95% CI: 255.62–261.36). Similarly, animals calving at 21 months of age had the lowest predicted mean 305-day protein yield (212.46 kgs; 95% CI: 210.32–214.60).

**Fig 4:**
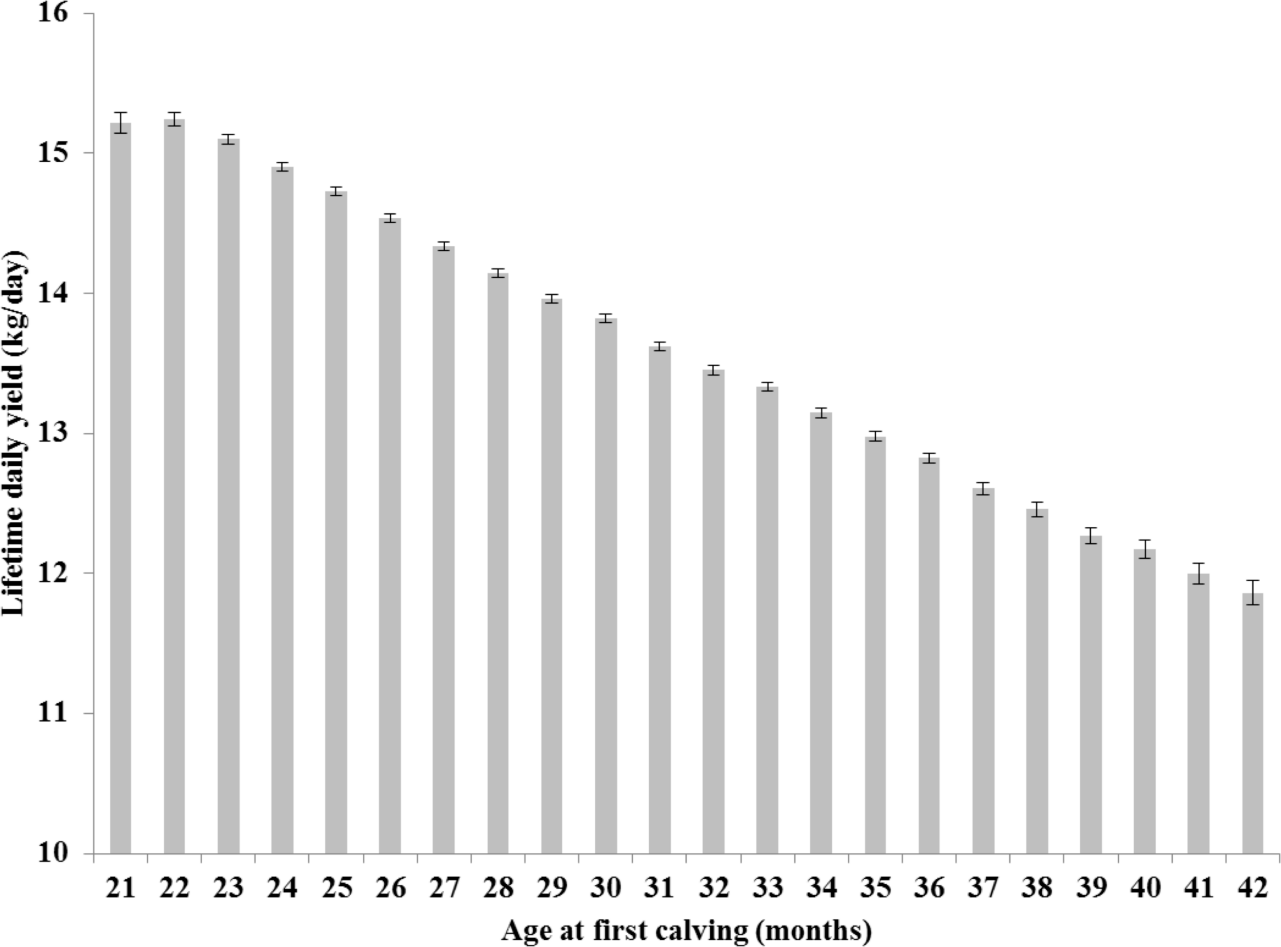
Age at first calving and recorded lifetime daily yield. Predicted means (± 95% confidence intervals) obtained from mixed effects multivariable regression modelling for recorded lifetime daily yield (milk kgs) by different age at first calving (AFC) class.

On the contrary, RLDY (kgs) increased as AFC decreased (see Fig 4). The 22-month age class are predicted to have the highest mean RLDY (15.25 kgs 95% CI: 15.16–15.35), significantly higher than those animals with an AFC of 24 months (14.92 kgs; 95% CI: 14.85–14.98) or greater. Primiparous cows within the 42 month AFC class are expected to produce significantly less milk than all other AFC classes (11.87 kgs, 95% CI: 11.7–12.04).

**Fig 5:**
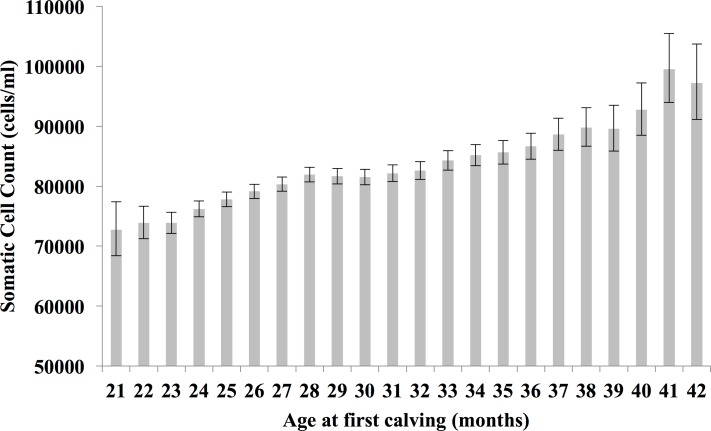
Age at first calving and first lactation somatic cell count. Back-transformed predicted means (± 95% confidence intervals) obtained from mixed effects multivariable regression modelling for first lactation (305 day) log somatic cell count (cells/ml) by different age at first calving (AFC) class.

Predictions relating to SCC are presented in Fig 5. Heifers with an AFC of 21 months are predicted to have the lowest 305-day lactation SCC (72,765.41; 95% CI: 68,427.08–77378.80), significantly lower than those heifers with an AFC of 26 moths (79,135.98; 95% CI: 77,948.90–80,341.14) or greater. The 41 month AFC class is predicted to have the highest mean SCC at 99,558 cells (95% CI: 93,952–105,498).

**Fig 6:**
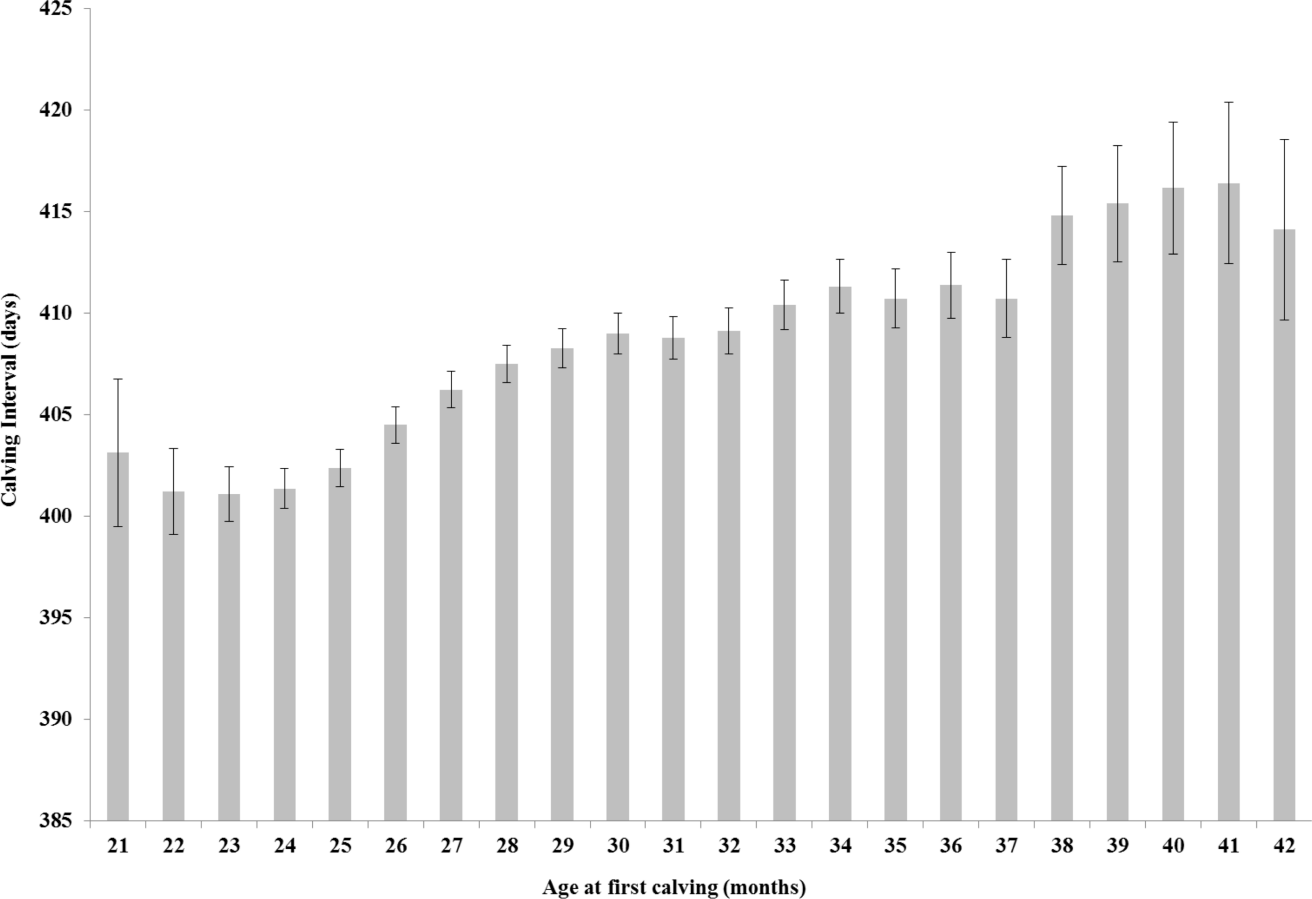
Age at first calving and calving interval. Back-transformed predicted means (± 95% confidence intervals) obtained from mixed effects multivariable regression modelling for calving interval (days) by different age at first calving (AFC) class.

When examining fertility performance (Fig 6) the 23 month AFC class is predicted to have the lowest calving interval (401.1 days; 95% CI: 399.8–402.4 days), significantly lower than those heifers with an AFC class of 26 and greater. The highest calving interval is predicted for the 41 month AFC class (416.4 days; 95% CI: 412.5–420.4 days), significantly higher than those heifers with an AFC of 33 months or less.

The predicted probabilities (with 95% confidence intervals) of calving for a second time by AFC class (months) are shown in Table 1. Animals calving at a younger age were significantly more likely to calve for a second time compared to animals calving for the first time at an older age.

**Table 1:** Predicted probabilities (with 95% confidence intervals) of calving for a second time by Age at First Calving (AFC) class (months).

AFC	Predicted Probability	95% Confidence Interval
21	0.829	0.811–0.848
22	0.837	0.826–0.848
23	0.825	0.818–0.833
24	0.826	0.820–0.832
25	0.817	0.812–0.822
26	0.807	0.802–0.812
27	0.804	0.798–0.809
28	0.793	0.787–0.798
29	0.785	0.780–0.791
30	0.782	0.776–0.788
31	0.772	0.765–0.778
32	0.762	0.755–0.769
33	0.754	0.746–0.761
34	0.748	0.740–0.756
35	0.737	0.728–0.746
36	0.726	0.717–0.736
37	0.720	0.709–0.731
38	0.699	0.686–0.712
39	0.689	0.673–0.704
40	0.690	0.672–0.707
41	0.666	0.645–0.687
42	0.649	0.626–0.672

## Discussion

We show here that only 20.1% of the studied population of UK dairy heifers (79,503) calved at 23 to 25 months of age and that 40.9% (162,157) were 30 months or older at their first calving. The average AFC in this study (29.1 months) is very similar to that reported in another, smaller scale, UK study in which the average AFC was 29.6 months with 35.9% of heifers calving at an AFC >30 months [13]. As this dataset includes all UK pedigree milk recorded Holstein and Holstein Friesian heifers calving in a three-year period it provides a comprehensive representation of UK performance with regard to AFC. Whilst AFC performance is similar to that determined in other studies [5,11,12,15] it is suboptimal when compared to the 24-month industry target. Our study is not without limitations though. In order to include animals with at least three complete lactations we had to study animals that calved for the first time in 2006–2008. Therefore, our data may not be an accurate representation of the current AFC in the UK Holstein population. Additionaly, a significant number of farms in this study were only contributing with less than 10 animals and this may have affected some the presented results.

Primiparous cows calving at 21 months were predicted to produce significantly less milk than other AFC classes and this is in line with other studies [4,12,16,17]. Martinez (1983) [18] proposed that a reduction in AFC may be associated with an increased risk of dystocia. Reduced production could be a direct consequence of this [19]. It is also highly likely that animals calving at younger age have a lower first lactation yield because they are partitioning greater amounts of energy towards growth during their first lactation comparing to animals calving at older ages and when they have reached a mature size [20,21]. Studying these phenomena was however beyond the scope of our study. The predicted average yield of the 24 month AFC class is 7,165 litres, some 609 litres less than the 36 month AFC class. It is however unlikely that the revenue from the potential extra milk realised by delaying calving outweighs the additional associated rearing costs (especially in light of our results associated with RLDY). The increase in overall weight of fat and protein produced with increasing AFC mirrored the trends observed with milk yield.

On the contrary, younger heifers in our study had the highest RLDY. This was consistent with previous studies which found an AFC between 22 and 26 months of age maximised lifetime daily yield, number of lactations and the number lactating days in an animal’s life [12]. A recent study from the US noted that heifers calving at 21 and 22 months of age produced 510 and 632 kgs, respectively, more of milk over their lifetime when compared to those calving at 24 months of age [22].

An association between increased AFC and increased SCC was found to be significant. To the best of our knowledge this is the first time such an association is being reported. The manifestation of subclinical mastitis in lactating primiparous cows can result from infection acquired before calving. Therefore one possibility is that an increased period of pre-calving exposure to mastitis pathogens in older heifers may account for the higher SCC’s seen [23]. A high early lactation or average lactation SCC is associated with an increased likelihood of culling [24–26] and a reduction in lactation milk yield [27,28]. Whilst SCC is used here as a measure of subclinical mastitis the close relationship between SCC and clinical mastitis that exists [29,30] dictates that a lower AFC is also likely to be consistent with a reduced clinical mastitis incidence.

AFC was found to be associated with calving interval between lactation 1 and 2. The lowest predicted mean calving interval was 401 days and was predicted for the 23 month AFC class. Similarly, in a recent large study of the performance of heifers calving for the first time in Belgium, the lowest mean calving interval was 410 days and was recorded for the 22–26 month AFC class [12]. Heifers with a low AFC have expressed good fertility in order to conceive and calve at a young age. This could then be associated with subsequent good fertility and a low calving interval. This trend is mirrored in an Irish study that determined that heifers calving at 25–26 months of age recorded the lowest subsequent calving interval [31].

Whilst calving interval represents a useful tool in terms of monitoring fertility, limitations do exist. The most obvious is that it fails to take into account those animals that do not calve again. As a result, it is important to consider the percentage of animals by AFC class known to have calved again for a second time. This varied between 75.6% (42 month AFC class) and 89.2% (22 month AFC class) for heifers that had completed a qualifying first lactation. These are higher values than those observed in other studies [12,32], but are comparable to a recent UK study [13] which found that mean culling rate for first lactation heifers was 16.9% (across all AFC classes). In keeping with other studies [12,13] we demonstrated here that a low AFC did not appear to affect survivability from lactation one to two. On the contrary, an increasing AFC was clearly associated with a reduced likelihood of calving for a second time. Several studies have highlighted the increased risk of culling associated with a high AFC [13,17,33,34]. A recent UK study concluded that a decrease in AFC from 27 to 24 months of age is associated with a 10% reduction in the odds of removal from the herd [35]. Furthermore, when considering survival beyond lactation two, it has been shown that as AFC increased beyond 25 months fewer animals reached lactation three [8].

Our results overwhelmingly support a lower AFC as a target. Animals calving at a younger age were found to have higher lifetime daily milk yield, lower SCC, lower calving intervals and increased likelihood of making it to the second lactation. Economic modelling such as that carried out by Pirlo (2000) [5] is however needed in order to better investigate the economics of these relationships. Lifetime profit declined from approximately 2,600,000 ($2,363.6) to 800,000 Won ($727.3) when age at first calving increased from 680 (22.3 month) to 1,000 d (32.8 month) in a recent study conducted on a large population of South Korean Holsteins [36]. On the contrary, a different research group concluded that achieving low AFC will not always be the most profitable approach, and that this will depend upon farm-specific herd management [37]. Such economic analyses were however beyond the scope of our study.

The management factors associated with the successful manipulation and attainment of a low AFC require a holistic approach and skillset that is likely cross transferable to other areas of on farm management such as milk production, SCC and mastitis control and fertility. As a result, the improved performance of younger primiparous cows that this study has described could be considered as an inevitability of superior management within the single farm. However, we have accounted for farm and sire (in an attempt to account for possible genetic effects) effects in our analysis and heifers calving between 22 and 25 months of age still outperformed their older counterparts with regard to lifetime production, fertility and mastitis performance.

## Conclusion

A lower age at first calving was shown here to be associated with better reproductive performance, increased survivability and decreased SCC. First lactation milk production was lower among younger heifers but lifetime daily milk yield was significantly increased.
